# DATS, the data tag suite to enable discoverability of datasets

**DOI:** 10.1038/sdata.2017.59

**Published:** 2017-06-06

**Authors:** Susanna-Assunta Sansone, Alejandra Gonzalez-Beltran, Philippe Rocca-Serra, George Alter, Jeffrey S. Grethe, Hua Xu, Ian M. Fore, Jared Lyle, Anupama E. Gururaj, Xiaoling Chen, Hyeon-eui Kim, Nansu Zong, Yueling Li, Ruiling Liu, I. Burak Ozyurt, Lucila Ohno-Machado

**Affiliations:** 1 University of Oxford, Oxford e-Research Centre, 7 Keble Road, Oxford OX1 3QG, UK; 2 Inter-university Consortium for Political and Social Research, University of Michigan, PO Box 1248, Ann Arbor, Michigan 48106-1248, USA; 3 University of California San Diego, 9500 Gilman Dr, La Jolla, California 92093, USA; 4 The University of Texas Health Science Center at Houston, 7000 Fannin St, Houston, Texas 77030, USA; 5 National Cancer Institute, National Institutes of Health, 9609 Medical Center Dr, Rockville, Maryland 20850, USA

**Keywords:** Computational biology and bioinformatics, Data processing

## Abstract

Today’s science increasingly requires effective ways to find and access existing datasets that are distributed across a range of repositories. For researchers in the life sciences, discoverability of datasets may soon become as essential as identifying the latest publications via PubMed. Through an international collaborative effort funded by the National Institutes of Health (NIH)’s Big Data to Knowledge (BD2K) initiative, we have designed and implemented the DAta Tag Suite (DATS) model to support the DataMed data discovery index. DataMed’s goal is to be for data what PubMed has been for the scientific literature. Akin to the Journal Article Tag Suite (JATS) used in PubMed, the DATS model enables submission of metadata on datasets to DataMed. DATS has a core set of elements, which are generic and applicable to any type of dataset, and an extended set that can accommodate more specialized data types. DATS is a platform-independent model also available as an annotated serialization in schema.org, which in turn is widely used by major search engines like Google, Microsoft, Yahoo and Yandex.

## Introduction

The biomedical and healthCAre Data Discovery Index Ecosystem (bioCADDIE, https://biocaddie.org) is an international joint effort of researchers, informaticians, data scientists, IT professionals, governmental agencies, services providers, publishers and the NIH to facilitate the discovery of available biomedical datasets that are spread across different databases, repositories and on the cloud, through the development of a Data Discovery Index (DDI). The NIH BD2K^1^ DDI Consortium is a direct response to the key recommendations made in 2012 by the Data and Informatics Working Group (https://acd.od.nih.gov/diwg.htm), set by the Advisory Committee to the Director of the NIH, that requested the development of a catalog of biomedical data. bioCADDIE started effectively when strategies and activities needed to implement the DDI were released as a white paper in 2015 (ref. 2).

The DDI prototype, named DataMed (https://datamed.org), was first launched in 2016. Currently, it enables browsing and searching over 60 biomedical data sources (as of March 2017), getting recommendations on datasets that best satisfy the users’ specific interests, preferences, and needs. Also the ISA-formatted metadata^3^ associated to each Data Descriptor article in *Scientific Data* (http://scientificdata.isa-explorer.org) are also indexed and available in the current DataMed release.

DataMed focuses on Findability (F) and Accessibility (A) of datasets. Along with Interoperability (I) and Reusability(R), F&A compose the four elements of the widely-endorsed FAIR principles^4^, which put a specific emphasis on enhancing the ability of individuals and machines to automatically discover and (re)use digital objects, throughout their life cycle. DataMed’s architecture encompasses: (i) a repository ingestion and indexing pipeline, which maps the disparate metadata from the diverse repositories, into the unified DATS model; (ii) a terminology server to ensure metadata consistency at a semantic level; and (iii) a web application based on the search engine, which uses the discovery index for locating the appropriate datasets from the set of repositories, and also the terminology server to expand user queries.

DATS metadata elements are used for indexing and searches in DataMed, and are presented as two modules: a core and extended set of elements. The DATS model has been specifically named to echo the JATS metadata elements and format (https://jats.nlm.nih.gov) required by PubMed (https://www.ncbi.nlm.nih.gov/pubmed) to index publications. Like JATS, the core DATS elements are generic and applicable to any type of dataset. As described in the DataMed paper in *Nature Genetics*^5^, the extended DATS includes additional elements. Some of these elements are specific for life, environmental and biomedical science domains and can be further extended as needed. The similarity and complementarity between DataMed and PubMed are not a simple convenience: it reflects the ecosystem of interconnected resources that are necessary to support and enable biomedical research as a digital research enterprise, known as the NIH Commons^6^. As the Data Science culture grows, all digital research outputs (not just publications and datasets, but also software, workflows, training materials, standards, etc.) must be FAIR. The goal is to empower scientists to fully utilize existing outputs to accelerate their research.

Throughout the project, the bioCADDIE team has undertaken a variety of community-driven activities to design and deliver DataMed; a detailed description of our journey and the resulting implementation are the focus of the complementary DataMed article. Here we focus on presenting the DATS model, its design principles, the methodologies used, the serializations and implementations to date.

## Results

The DATS model describes the metadata elements and the structure for datasets, and powers DataMed’s ingestion and indexing pipeline, as well as its search functionality. The model also follows the FAIR best practices for data management, and the recently released ‘Candidate Recommendations on Data on the Web Best Practices’ (https://www.w3.org/TR/dwbp) by the Data on the Web Best Practices Working Group (WG) under the World Wide Web Consortium (W3C). Both community practices support that data on the web should be discoverable and understandable by humans and machines; and that such usage should also be discoverable and the efforts of the data publishers recognized.

The work has been driven and executed by the bioCADDIE team with the input and feedback of international researchers, service providers and knowledge representation experts. Community participation was coordinated via the bioCADDIE’s Descriptive Metadata WG3 and the Accessibility Metadata WG7; participants are listed in the Acknowledgement section. The Metadata WG is a joint activity with a wider Metadata WG—encompassing other BD2K centers of excellence—and closely connected to the NIH BD2K Center for Expanded Data Annotation and Retrieval^7^ and ELIXIR activities in Europe (https://www.elixir-europe.org). Currently, the NIH National Library of Medicine (NLM) is exploring DATS and its possible role in ongoing efforts to make a broader range of biomedical data more readily discoverable.

In August 2015, the first version (v1.0) of the DATS specification was released^8^, with the model available as machine readable JSON (http://json-schema.org) schemata and with several examples. This initial model and each of the following versions were tested by the DataMed development team, with a variety of data sources, and reviewed by the bioCADDIE’s Descriptive Metadata WG3 members and the larger community. As a result of this iterative process, specifications v1.1 (ref. 9), v2.0 (ref. 10) and v2.1 (ref. 11) were released in March, June and September 2016, respectively. The specification v2.0 represents a further evolution of the model, including the additional metadata elements produced by the Accessibility Metadata WG7, a set of JSON-schemas and JSON examples, and a Schema.org-annotated context file. The DATS v2.1 also includes edits based on feedback from a first bioCADDIE repository workshop and a Schema.org-annotated JSON-LD (http://json-ld.org) serialization. The current version v.2.2 (ref. 12) includes support for temporal and spatial extension. All specifications, serializations and examples are freely available from the bioCADDIE Github repository (https://github.com/biocaddie/WG3-MetadataSpecifications), where enquiries and feedback can be submitted and tracked. Each release is also packaged and released via Zenodo^8,9,10,11,12^ to receive a unique identifier for citation purpose and ensure long term preservation.

The following sections provide a description on the current DATS model v2.2.

### Scope and coverage: Focus on discoverability

First and foremost, it is important to clarify that DATS has not been designed to perfectly represent each and every step of an experiment; this level of detail and metadata are left to the specific repositories that DataMed works with to index. The scope of DataMed is discoverability: DATS provides enough metadata descriptors to help researchers find and access datasets they need and that are stored across a variety of repositories.

Developed iteratively, the model is the result of three complementary approaches, detailed in the Methods section; briefly: (i) a review of existing meta-models, (ii) an analysis of the use cases (top-down), and (iii) a mapping of existing metadata schemas (bottom-up), to find convergences and common metadata elements. Such a combined approach has been necessary to define the appropriate boundaries and level of granularity: which queries will be answered in full by the DataMed prototype, which only partially, and which queries are out of scope.

The DATS model is designed around the *Dataset* element, catering for any unit of information stored by repositories. *Dataset* covers both (i) experimental datasets, which do not change after deposition to the repository, and (ii) datasets in reference knowledge bases, describing dynamic concepts, such as ‘genes’, whose definition change over time. To implement the concept that DataMed is built as part of an interlinked ecosystem of resources, the *Dataset* element is linked to other digital objects, which are the focus of other indexes; specifically *Publication* (e.g., PubMed), *Software* (e.g., BD2K Aztec: https://aztec.bio), *DataRepository* and *DataStandard* (e.g., BioSharing^13^, https://biosharing.org). The latter is especially important in the light of the vast swathes of data that still remain locked in esoteric formats, are described using *ad hoc* or proprietary terminology or lack sufficient contextual information. Knowing if a repository uses open community standards to harmonize the reporting of its different datasets, will provide researchers with some confidence that these datasets are (in principle) more comprehensible and reusable.

Key information about the *Dataset* element is its accessibility, which is represented by the *Access* metadata element that encompasses information on authorization, authentication, and access type. Many types of data in the biomedical domain are restricted to protect confidential information about human subjects. Researchers want to know which data sets are readily available and which ones require prior approval, institutional data use agreements, and security clearances. The *DatasetDistribution* element, linked to the *DataRepository, DataStandard* and *Access,* also informs researchers about which data can be accessed directly by machines through an application programming interface (API).

### Modular model: Domain-agnostic core with extended elements

**Figure 1:**
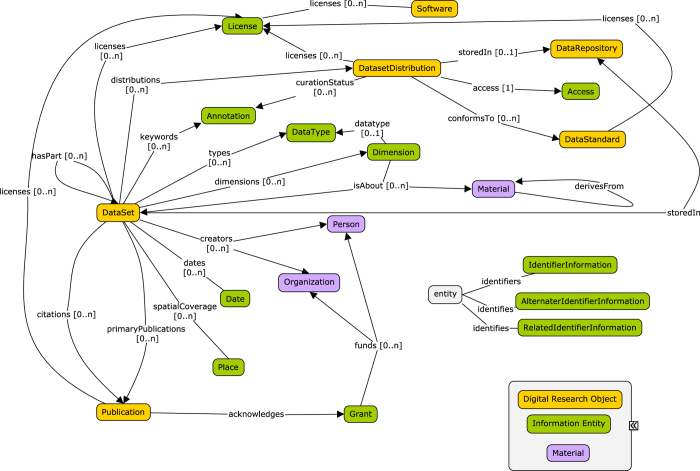
A schematic overview of the DATS core elements, their types and relations.

**Figure 2:**
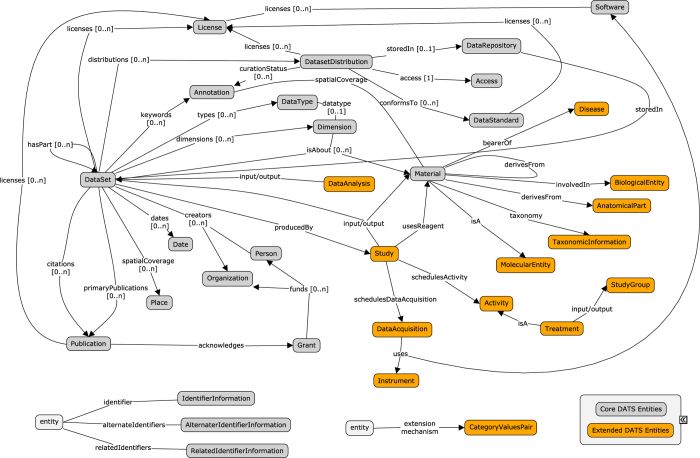
A schematic overview of the DATS core and extended elements, their types and relations.

**Figure 3:**
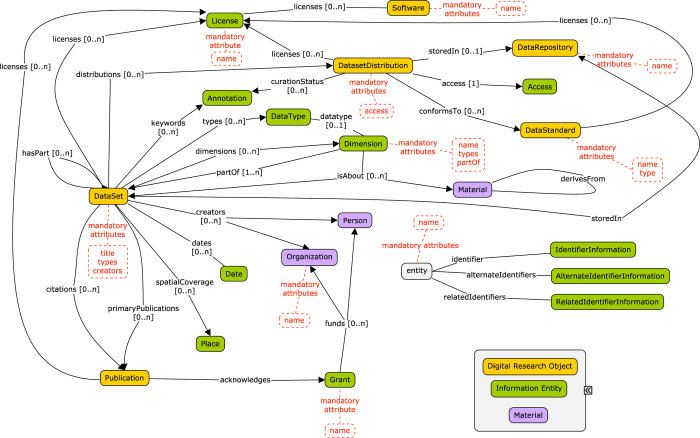
A schematic overview of the DATS core entities and theirs few properties with requirement level ‘MUST’.

The Google JSON style guide (https://google.github.io/styleguide/jsoncstyleguide.xml) has been used to name relevant elements in the DATS model; the requirement levels, indicating what elements are compulsory or desired in the specification, follow the standard RFC 2,119 (https://tools.ietf.org/html/rfc2119). The descriptors for each metadata element (or Entity), include: Property (attributes describing the Entity or relating the entity with other entities), Definition (of each Entity and Property), Value(s) (allowed for each Property). In both core and extended DATS, Entities are not mandatory, but applicable only if relevant to the dataset to be described; when an Entity is used, only some of its Properties are defined as mandatory. For example, for the 18 entities of the core there are only a total of 10 mandatory properties. An overview of the core and extended entities, their types and relations are shown in Figs 1 and 2, respectively; Fig. 3 provides a view of the core entities highlighting the few properties defined as mandatory. DATS has been designed to specifically drive discoverability: to find and access datasets via key metadata descriptors. DATS is not meant to perfectly model an experiment, which is the scope of many repository-level models and schemas. The DATS model may appear quite detailed in places as a consequence of (i) the combined approaches used to identify the required metadata elements (see section 2), and (ii) the attempt to aim for the maximum coverage of use cases with minimal number of metadata elements. Nevertheless, it is anticipated that not all use cases can be fulfilled, and that it is difficult to foresee all types of data sources the DataMed prototype should retrieve information from.

Like the JATS, the DATS core elements are generic and applicable to any type of datasets, and cover basic information, for example: what the dataset is about (using the *Material* entity), how it was produced and which information it holds (*DataType*, *Information*, *Method*, *Platform*, *Instrument*), where it can be found (*DatasetDistribution*, *Access*, *Licence*), when it was released and by whom was it created and funded (*Person*, *Organization* and their roles, *Grant*). A set of these DATS core elements also map to the minimal metadata requirements for repositories’ landing pages to support data citation; this work was done by the Repositories Early Adopters Expert Group^14^, part of the Data Citation Implementation Pilot (DCIP) project, an initiative of FORCE11 (https://www.force11.org) and bioCADDIE. The DATS extended elements have been created to progressively accommodate domain-specific metadata for more specialized data types. The current extended set is tailored to DataMed use cases, and therefore specific for life, environmental and biomedical science domains, but it can be further extended as needed.

### Serializations: Schema.org and bioschemas

Currently the core and the extended DATS entities are available as machine readable JSON schemata, and as a JSON-LD serialization annotated with Schema.org (http://schema.org); serializations in other formats can also be done, if needed. Schema.org is a collaborative, community activity with a mission to create, maintain, and promote schemas for structured data on the Internet, on web pages, in email messages, and beyond. Sponsored by Google, Microsoft, Yahoo and Yandex, Schema.org is already used by over 10 million sites to markup their web pages and email messages. Therefore, anchoring the metadata model to such a potentially powerful vocabulary was one of the early design decisions, outlined in the bioCADDIE white paper.

As Schema.org is a generic vocabulary, its use in the life and biomedical areas requires some extensions. Gaps in coverage have been identified during the annotation process: notifications of these gaps have been submitted to the Schema.org Github tracker. A file mapping DATS to Schema.org elements is available as part of the DATS model v2.2 release; this may be subject to change as current Schema.org elements evolve. In addition, members of the bioCADDIE team are working to coordinate extensions to Schema.org for datasets by: communicating with the WC3 Healthcore and Schema Vocabulary Community Group (https://www.w3.org/community/schemed), focused towards clinical studies; and participating and leading activities under the Bioschemas umbrella (http://bioschemas.org), which includes major data repositories, BD2K and ELIXIR resources and is set to cover other digital objects beyond data.

Ultimately, the bioCADDIE team will deliver a Schema.org-annotated DATS model or profile, by packaging relevant Schema.org (and/or Bioschemas extended) vocabularies that are relevant to the DATS elements. By delivering this Schema.org DATS profile, web-based DataMed will benefit from: an increased visibility by major search engines and tools, increased accessibility via common query interfaces, and possibly, an improvement in search ranking. Conversely, data repositories indexed by bioCADDIE will also get the benefit from being more visible to search engines beyond DataMed.

## Adoption

Developed iteratively with the input and feedback of a community of international researchers, service providers and knowledge representation experts, DATS is currently implemented by the bioCADDIE team in DataMed; this is the intended and primary scope for this metadata model. In the first instance, the bioCADDIE team has mapped the target repositories’ model to DATS to inform the implementation of data harvesting converters. This has also enabled the team to test the DATS model in real life, refine it, deliver documentation and create examples that have already been used to enable prospective users to export the relevant metadata for indexing in DataMed. To further the uptake, the bioCADDIE team has gone to great lengths and released mappings of DATS to a number of existing generic and widely used schemas, including Schema.org, and domain specific repository schemas; this is available as part of the DATS model v2.1 release. As the work progresses under Bioschemas, a Schema.org DATS profile will also be released, via the Github and Zenodo platforms.

Especially notable are our successful collaborations with other data aggregators and service providers that are working to implement DATS. These include—but are not limited to: the Inter-university Consortium for Political and Social Research (ICPSR), the world's largest archive of digital social science data and one of the NIH-supported repositories (https://www.nlm.nih.gov/NIHbmic/nih_data_sharing_repositories.html); the NIH BD2K OmicsDI^15^, a data discovery index for proteomics, genomics and metabolomics datasets; the NIH Federal Interagency Traumatic Brain Injury Research (FITBIR, https://fitbir.nih.gov), an informatics system to share data across the entire TBI research field; ImmPort (http://www.immport.org), an informatics system supporting the NIH mission to share data, focused on the immunology data; and DataCite (https://www.datacite.org), a global non-profit organisation that supports the creation and allocation of Digital Object Identifiers (DOIs) for research data and accompanying metadata. Another example of use is provided by the NIH BD2K CEDAR metadata authoring tool that works to provide a DATS-compliant template to help researchers to describe and expose their datasets, which are not yet in public repositories, to indexing in DataMed.

**Figure 4:**
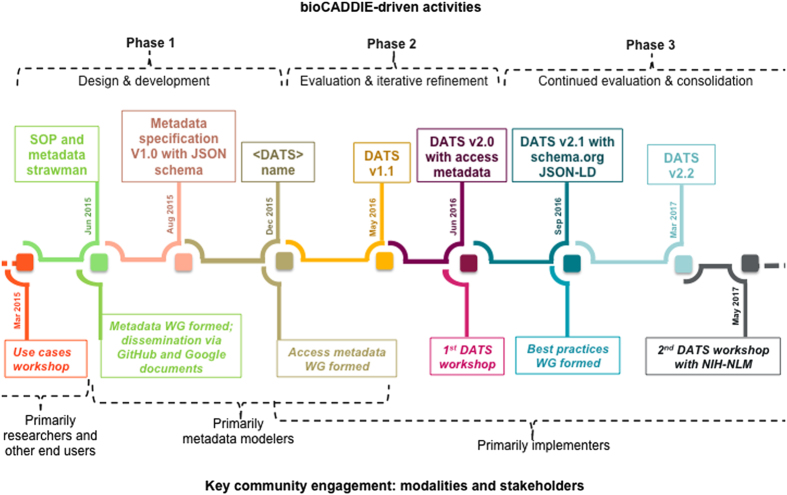
Overview of the development process.

Figure 4 provides an overview of the development process, including key deliverables and community engagement. To continue these collaborations and foster new ones, the bioCADDIE team has also set up a WG on Standards-driven Curation Best Practices to: (i) assist repositories in creating DATS metadata from the information that they hold; (ii) draw lessons about data curation from experiences of developing data harvesting converters in DataMed; and (iii) encourage best practices that make data FAIR. Furthermore, the NIH NLM is also exploring DATS and its possible role in ongoing efforts.

## Discussion

A digital ecosystem, such as the NIH Commons, consists of objects (publications and datasets, but also software, code, workflows, training materials, tools, standards, etc.) that are indexed in a consistent fashion, with information related to their origin, contents, and availability. These objects must be findable, accessible, interoperable, and reusable, according to the FAIR principles; this is achieved through a series of technical and social processes involving researchers, informaticians, data scientists, IT professionals, governmental agencies, services providers and publishers.

In this article, we described one portion of the infrastructure, namely the model for the dataset object of this ecosystem. Through a process in which we identified potential use cases and common elements across existing metadata models, we were able to produce a metadata model that is being implemented by the BD2K DataMed prototype, as well as used by other initiatives. The model is expected to continue to evolve with input from the community, and to be used directly by repository managers and data producers to map from their existing metadata. As the model matures, we anticipate new tools and APIs will be developed and the boundary between the shallow indexing intended for DataMed and the deep indexing provided by the repositories and community aggregators to be crossed seamlessly.

## Methods

The DATS model was developed given the following three considerations and approaches.

1. A variety of data discovery initiatives exist or are being developed; although they have different scope, use cases and approaches, the analysis of their metadata schemas is valuable. Several meta-models for representing metadata were reviewed to determine essential items: these have more specific aims and different use cases than the intended capability of DataMed. The reviewed schemas and models are listed in a dedicated BioSharing Collection for bioCADDIE (https://www.biosharing.org/collection/bioCADDIE).
2. Identification of the initial set of metadata elements was based on: (i) analyses of use cases (a *top-down* approach); and (ii) mapping of existing metadata schemas (a *bottom-up* approach), to find convergences and common metadata elements.
3. Use cases have been guiding elements throughout the development process, in order to define the appropriate boundaries and level of granularity for the model. The use cases have been: (i) collected at the bioCADDIE Use Cases Workshop, (ii) extracted from the bioCADDIE White Paper, (iii) provided by the NIH, and (iv) submitted by the community. From these use cases, a set of ‘competency questions’ were derived; these defined the questions that we want DataMed to answer in full, only partially, and which are out of scope. The questions were abstracted, key concepts were highlighted, color-coded and binned in entities, attributes and values categories, to be easily matched with the results of the *bottom-up* approach.

The DATS v2.2 specification^12^ also contains: (i) key examples of competency questions where the concepts highlighted have been binned in entities, attributes and values categories; (ii) an appendix document detailing the DATS elements that are associated to relevant use cases and/or to the existing schema(s)/model(s) used in the development process, to justify their relevance and provenance; and (iii) a file mapping generic metadata schemas and key life science-specific ones.

## Additional Information

**How to cite this article:** Sansone, S.-A. *et al.* DATS, the data tag suite to enable discoverability of datasets. *Sci. Data* 4:170059 doi: 10.1038/sdata.2017.59 (2017).

**Publisher’s note:** Springer Nature remains neutral with regard to jurisdictional claims in published maps and institutional affiliations.
